# Tilescope: online analysis pipeline for high-density tiling microarray data

**DOI:** 10.1186/gb-2007-8-5-r81

**Published:** 2007-05-14

**Authors:** Zhengdong D Zhang, Joel Rozowsky, Hugo YK Lam, Jiang Du, Michael Snyder, Mark Gerstein

**Affiliations:** 1 Department of Molecular Biophysics and Biochemistry, Yale University, New Haven, CT 06520, USA; 2 Interdepartmental Program in Computational Biology and Bioinformatics, Yale University, New Haven, CT 06520, USA; 3 Department of Computer Science, Yale University, New Haven, CT 06520, USA; 4 Department of Molecular, Cellular and Developmental Biology, Yale University, New Haven, CT 06520, USA

## Abstract

We developed Tilescope, a fully integrated data processing pipeline for analyzing high-density tiling-array data . In a completely automated fashion, Tilescope will normalize signals between channels and across arrays, combine replicate experiments, score each array element, and identify genomic features. The program is designed with a modular, three-tiered architecture, facilitating parallelism, and a graphic user-friendly interface, presenting results in an organized web page, downloadable for further analysis.

## Abstract 2

Tilescope is a fully integrated and automated new data-processing pipeline for analyzing high-density tiling-array data.

## Rationale

Microarray technology is now more accessible than ever before. Thanks to its unrivaled capability to carry out a very large number of parallel quantitative measurements, this technology has been widely applied since its emergence in the early 1990s [1,2] to systematic studies of various biological phenomena, ranging from differential gene expression, to DNA copy number polymorphism, and to transcription factor binding.

Traditional microarrays, constructed by mechanically depositing or printing PCR products, typically of approximately 1 Kb in length, in a dense matrix on a glass slide, have been successfully used in numerous studies and have become prevalent in the research field. Many computer programs and software tools, including free software packages, such as ExpressYourself [3] or MIDAS [4], are available to process and analyze the data sets generated in such studies. However, limited by its manufacturing methodology, traditional microarrays are not amenable for systematic coverage of large genomes or even some large genomic regions. To fully realize the parallel-measurement potential of microarray technology, the current trend is to present large genomic regions (for example, ENCODE regions or a complete human chromosome) or even an entire genome on one or several microarrays in an unbiased fashion by using oligonucleotides (that is, tiles) uniformly sampled from presented genomic sequences. Recent technology breakthroughs [5,6] made it possible for such oligonucleotides, typically of 25-60 base-pairs (bp) in length, to be chemically synthesized directly on the microarray slides in a very high density (up to 6.6 million elements in less than 2 cm^2^). Such oligonucleotide tiling microarrays, which give unprecedented genomic coverage and resolution, can be used for genomic studies of gene expression [7-10], chromatin immuno-precipitation (ChIP-chip) [11], copy number variation [12], histone modification [13], and chromatin DNaseI sensitivity [14].

Like for any other nascent technologies, ready-to-use data analysis software packages for tiling array experiments are hard to find. Existing data processing software for traditional microarrays cannot be used since the considerably larger size and different nature of tiling array data require a new analysis approach [15]. Recently, a model-based method for tiling array ChIP-chip data analysis has been proposed [16]. Two other methods, based on curve fitting [17] and multi-channel combination [18], respectively, have also been developed for tiling array transcription data analysis. The excellent open-source Bioconductor software project [19] provides many sophisticated statistical methods written in R for microarray data analysis. However, as a software toolbox and a programming environment, it is rather difficult for non-programmers to use.

Here we present Tilescope, an automated data processing pipeline for analyzing data sets generated in experiments using high-density tiling microarrays. Suitable microarray data processing methods, either previously published elsewhere or newly developed, were implemented and made available conveniently in a single online software pipeline. It has a user-friendly interface and is freely accessible over the worldwide web. The software performs data normalization, combination of replicate experiments, tile scoring, and feature identification. We demonstrate the modular nature of the pipeline design by showing how different methods can be plugged in - at major data processing steps, such as normalization and feature identification, several methods are available to be chosen from depending on the nature of the data and the user's data-analysis goal. The program can process gene expression and ChIP-chip tiling microarray data. The results, presented in a clear, well organized manner, can be downloaded for further analysis.

## System implementation and user interface

Tilescope was entirely developed in Java. Java was chosen as the programming language because of its built-in threading capability and its excellent library support for graphic user interface and networking development. More importantly, it was chosen because of its object-oriented nature: the program code is organized into different coherent classes and, thus, it naturally modularizes the system, which greatly facilitates parallel system development and subsequent system updating, a *desideratum *for any software engineering project of non-trivial complexity.

As a web-accessible program system, Tilescope is composed of three connected components: an applet, a servlet, and a pipeline program. The applet is the graphical interface through which the user interacts with Tilescope. It is automatically downloaded and launched inside a Java-enabled web browser whenever the pipeline web page is browsed. Through the Tilescope applet, a user can upload array data files to the pipeline server, select appropriate pipeline parameters and methods, run the data processing program, and view or download analysis results. The applet, however, cannot run the pipeline program directly. Instead, it makes data processing requests to the servlet, a server program that acts as the proxy of the pipeline program on the web and communicates with the applet upon requests. The servlet, the central layer of Tilescope, runs two 'daemon' threads in the background to handle - that is, accept and schedule or reject based on the current system load - file upload or data processing requests, prepare the pipeline running environment, and initiate with user-specified parameters the back-end pipeline program, which carries out the heavy lifting - the actual data processing procedure. This modular design - the separation between the request handling and the data processing itself - enables the usage of a computer farm for parallel computing and multiple concurrent processing.

**Figure 1:**
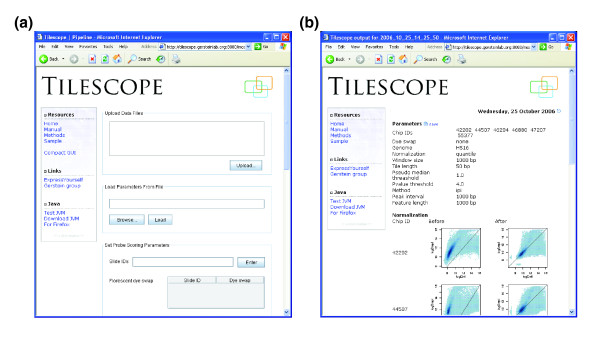
Screenshots of Tilescope. **(a) **The applet of Tilescope, the graphic user interface of the pipeline. **(b) **An example of the data analysis result web page.

On the web form of the Tilescope applet (Figure 1a), a user can either upload a parameter file, if available from a previous use of Tilescope, to have all parameters set accordingly in one easy step, or set parameters one by one manually, which is more likely to happen if an array data set is to be analyzed for the first time. The main body of the form was organized into two panels, one for setting the tile scoring parameters and the other for selecting the feature identification method, reflecting two main stages of data processing in the pipeline. After the pipeline program is started on the server, the users can monitor its progress through pipeline messages, which are constantly updated by the server throughout each pipeline run.

When data processing is done, a web page with analysis results will be presented to the user in a new browser window (Figure 1b). On the result web page, the parameters and methods that were used to analyze the data are summarized at the top, followed by log-intensity scatter plots for each array and log-intensity histograms for all arrays in the data set before and after normalization. These enlargeable plots enable the user to quickly identify any problematic arrays visually and subsequently exclude them from further consideration. Both tile maps with log-ratio and *P *value annotations and the feature list in various text formats can be downloaded for further processing and analysis. The feature list in regular tab-delimited text format gives the user the chromosome (or other genomic sequence ID), the genomic start and end coordinates, the log-ratio, the *P *value, and, if the tiled genome is specified, the upstream and downstream genes of each feature. If it is the human genome that is under investigation, Tilescope will also provide links to display identified features on custom tracks in the UCSC genome browser. Moreover, if the tiling array was designed from a previous human genome build (for example hg16, NCBI 34), Tilescope will also provide an additional feature list with the coordinates lifted over to the current human genome build (for example hg17, NCBI 35).

## Data processing in Tilescope

**Figure 2:**
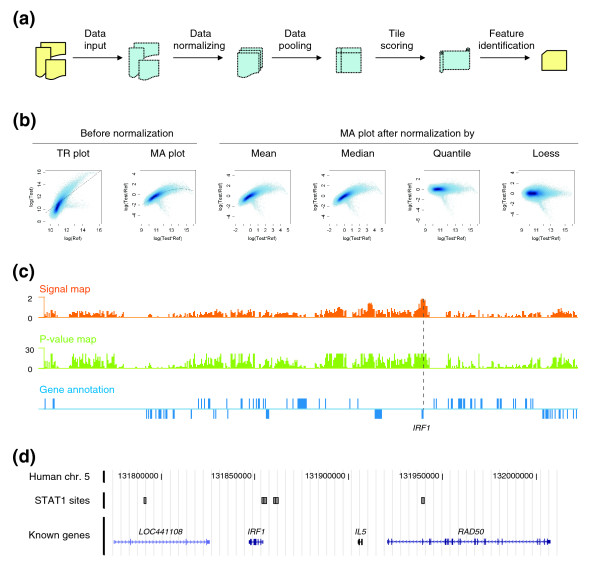
Tiling array data processing by Tilescope. **(a) **Flow chart of major data processing steps. Yellow icons represent data in user-accessible files, and blue ones data in the pipeline program memory. See main text for details. **(b) **Log-intensity scatter plots of a tiling array from the STAT1 experiment set before and after normalization by four different methods. The first panel is the log_2_*T *verses log_2_*R *plot before normalization, where *T *and *R *are test intensity and reference intensity, respectively. The gray line represents where these two log-intensities are equal. The second panel is log_2_(*T*/*R*) verses log_2_(*T*×*R*) plot (the MA plot) before normalization. The dependency of the log-ratio on the intensity, evinced by a fitted loess curve, is prominent in the data. The other panels are the MA plots of array data after mean, median, loess, or quantile normalization. They clearly show that the distribution of log-ratios is centered at zero by all normalization methods, but the intensity-specific artifacts in the log-ratio measurements are removed by only loess or quantile normalization and not by the mean- or median-based method. **(c) **Signal and *P *value maps of all tiles in the ENCODE ENm002 region. In this region, the tiles near the transcription start site of IRF1, a transcription factor known to be regulated by STAT1, give the strongest signals. **(d) **Tilescope-identified STAT1 binding sites at the 5'-end of IRF1 are shown on the custom track in the UCSC genome browser.

Tilescope processes the data in a sequential fashion using the major steps shown in Figure 2a. These steps can be approximately grouped into three stages: data input, tile scoring, and feature identification. Here, we use the data set from a ChIP-chip experiment of the transcription factor STAT1 to demonstrate how high-density tiling microarray data are processed by Tilescope. We compared features of Tilescope and several other programs that are explicitly applicable to high-density tiling microarray data, and the result is tallied in Table 1.

**Table 1:** Feature comparison between tiling microarray data analysis software*

	Tilescope	Bioconductor^†^	TAS^‡^	MAT^§^	TileMap
**Implementation**	Web	R packages	Standalone	Standalone	Standalone
**Graphic user interface**	√	×	√	×	×
**Intended usage**					
Transcription data	√	√	√	×	√
ChIP-chip data	√	√	×	√	√
**Applicable array platform**					
Affymetrix	√	√	√	√	√
NimbleGen	√	×	×	×	×
**Data normalization**					
Mean/median	√	~	√	/	×
Loess	√	~	×	/	×
Quantile	√	~	×	/	√
**Feature identification**					
Max gap and min run	√	~	√	/	√
Iterative peak identification	√ (new)	×	×	/	×
Hidden Markov model	√	~	×	/	√

*Only programs explicitly applicable to high-density tiling microarray data were considered. The websites of the compared programs are listed as follows: Tilescope at [35]; Bioconductor at [37]; TAS at [38]; MAT at [39]; TileMap at [40]. ^†^Strictly speaking, Bioconductor is not a ready-to-run program. It is a collection of software packages/libraries written in R. As a tool box, the analysis methods that it provides need to be written in an R program to run. ^‡^TAS is previously known as GTRANS. ^§^MAT standardizes the probe value through the probe model, which obviates the need for sample normalization. Comparison symbols used in the table: √, available; ×, not available; ~, available but need to be programmed; /, not applicable.

### Data input

The data input to Tilescope reside in tab-delimited text files generated by image analysis software. Currently, Tilescope recognizes data files in Affymetrix, Pair [20] and GFF [20,21] formats. Whenever available, GFF format is always recommended since it is a more standardized format and thus less problematic for processing. Although the aforementioned formats are not fully inter-compatible, they all provide the essential data, namely the chromosomes (or other genomic identifiers, such as Contig IDs and ENCODE region IDs), the genomic coordinate, and the fluorescence intensity (Tilescope automatically detects the base-two logarithm of intensity) for each array element.

We implemented Tilescope to support various formats of the input data file - the GFF format, the PAIR format, the NimbleGen format (POS + GFF/PAIR), and the Affymetrix format (BPMAP + CEL), and also developed a new algorithm (see Additional data file 1 for details) that can reduce the physical data file size, handle the data set in an organized manner, and enhance the performance of Tilescope.

### Data normalization

Unlike printed PCR arrays, the array elements (oligonucleotides) of a tiling microarray are directly synthesized on the array slide. Direct *in situ *synthesis creates morphologically uniform array elements, which, to a large degree, obviates the need for spot filtering, an imperative procedure for PCR microarray data analysis. Moreover, direct oligonucleotide synthesis makes it possible to have a very large number of spots in a small area (thus high-density). Miniature slide design of the tiling microarrays allows more uniform hybridization and, thus, greatly reduces the spatial heterogeneity in the probing conditions across the slide, a potentially severe problem suffered by PCR microarrays.

For each array in an experimental set, the relative contributions of the test and reference signals are compared. Ideally, if nucleic acid probes have equal concentration in the test and reference samples, the signals of the two dyes should be approximately equal (that is, the ratio of the two signals should be close to one for probes hybridizing to an equal degree in both fluorescence channels). In practice, the signals can be rather different due to different chemical properties of dyes and nonspecific or incomplete hybridization to the array. Normalization is used to compensate for these effects by - depending on what method is being used - either applying a scale factor to equalize signals from probes with unchanged concentration or imposing the same empirical distribution of signal intensities. We put together and implemented standard statistical methods that were described in various literature sources and made them conveniently available for tiling array data analysis. At present, Tilescope can normalize tiling array data by mean/median, loess, or quantile normalization (Figure 2b). These methods have also been implemented elsewhere, most notably in Bioconductor R packages. The mean/median and the loess normalization methods are both available in the 'marray' package. The 'affy' package contains another implementation of the loess and the quantile normalization methods. In addition, other publicly available software, such as TM4 [4] and TAS/GTRANS, provides some similar functionalities for array data normalization. These methods are summarized below with appropriate references.

#### Mean/median normalization

Normalization by mean or median [3], the so-called 'constant majority' methods, is based on the assumption that the majority of genes do not change their expression level in response to the experimental perturbation [22] or, more precisely, that the average or median gene expression level does not change under experimental perturbation. It is carried out by subtracting the mean or median of the base-two logarithm of the ratio of test to reference signal intensities from the log-ratio value of each tile on a single array. This procedure transforms the log-ratio distribution by centering it at zero. The mean of a probability distribution is its center of gravity, while the median divides it into two equal parts. In theory, they are different measures of the location of a distribution. In practice, however, because the mean and the median of the log-intensities from the probes on each array are often very close to each other, these two methods usually give very similar results. The advantages of these two methods include the easiness of their implementation and their robustness to the violation of the assumption - they remain applicable even in cases where up to 50% of probes have altered concentrations.

#### Loess normalization

Loess normalization [3,23,24] normalizes array data between channels and removes the intensity-specific artifacts in the log-ratio measurements simultaneously. Like normalization by mean or median, loess normalization is also performed on an array-by-array basis. For each array, Tilescope first uniformly samples 50,000 log-ratio values from the original data, and then performs the locally weighted regression on the sampled data. The dependency of the log-ratio on the intensity is removed by subtracting predicted log-ratio based on the loess regression from the actual log-ratio, and the new test and reference log-intensities after normalization are recovered from the residuals. The main disadvantage of Loess normalization is that the locally weighted regression is computationally intensive, and thus the necessity of using sampled data instead of the original, much larger data set. Since loess normalization is carried out for each array one by one, even after data sampling it remains expensive to use.

#### Quantile normalization

Unlike the normalization methods discussed above, quantile normalization [24] not only normalizes data between channels and across arrays simultaneously but also removes the dependency of the log-ratio on the intensity in one step. It imposes the same empirical distribution of intensities to each channel of every array. To achieve this, Tilescope first creates an *n*×2*p *(log) intensity matrix **M**, where *n *is the number of tiles on an array and *p *is the number of arrays in an experimental data set, and then sorts each column of **M **separately to give **M**_s_. Afterwards, it takes the mean across rows of **M**_s _and creates **M**_s_', a matrix of the same dimension as **M**, but where all values in each row are equal to the row means of **M**_s_. Finally, Tilescope produces the quantile-normalized (log) intensity matrix **M**_n _by rearranging each column of **M**_s_' to have the same ordering as the corresponding column of **M**. Quantile normalization is fast and has been demonstrated to outperform other normalization methods [24]. Thus, it is the default normalization method used by Tilescope.

### Tile scoring

Some arrays are designed to tile genomic sequences of both strands and most array experiments are conducted in replicate. To facilitate subsequent data processing, Tilescope pools the normalized log-ratios of all tiles on every array into a matrix and sorts them based on the tiles' genomic locations regardless of which strand they come from. (Thus, to use Tilescope to process strand-specific data, the user needs to parse data from plus-stranded tiles and minus-stranded tiles into two separate files and process them separately. This limitation will be addressed in the next version of Tilescope.) At the tile scoring step, the program identifies tiles that exhibit differential hybridization. Depending on the nature of the experiment, these tiles ultimately correspond to genes whose expression levels have changed or the locations of transcription factor binding sites (TFBSs).

Compared with traditional PCR arrays, tiling arrays accommodate a much larger number of array elements, which are *in situ *synthesized oligonucleotides, typically dozens of nucleotides long. However, there is a trade-off for better coverage of the genome: as the average length of the array elements gets smaller, the variance of data increases due to the rise of the relative magnitude of random noise and the possibility of cross-hybridization and sequence artifacts. To deal with this problem, Affymetrix used a different method to score (one-channel) tiling arrays [10,25] than the one used for PCR arrays; instead of considering each tile across array replicates separately, they used a sliding window around each tile to incorporate the hybridization intensity of its neighboring tiles. In our implementation of this method in Tilescope, we modified it by adding a nonparametric statistical test to assess the significance of the intensity difference between the test and the control samples at each tile. This extension enables us to score each tile using two different criteria. Moreover, we also adapted the original method to NimbleGen two-channel tiling arrays data, which in effect significantly increased the usability of this method.

For each tile, given its neighboring tiles across replicates, Tilescope calculates the pseudo-median log-ratio value as its signal. The pseudo-median (that is, the Hodges-Lehmann estimator) of the log-ratio is a nonparametric estimator of the difference between the logged intensities of the test sample and those of the reference sample. It is calculated for each tile using a sliding window. The tiles from all arrays in a sliding window are first collected into a tile set, and the pseudo-median is calculated for this window as:

*S *= median[ (log-ratio_*i *_+ log-ratio_*j*_)/2 ]

from all (*i*, *j*) pairs of tiles in the tile set. As a nonparametric estimator, pseudo-median is less susceptible to distributional abnormalities (such as skewness, unusual kurtosis, and outliers).

Due to the small sample size in each sliding window, whether the intensity distribution is normal or not in a given window cannot be reliably assessed. Without making the normality assumption about the intensity distribution, Tilescope uses the nonparametric Wilcoxon signed-rank test [26] to compare the test with the reference signal intensities and quantifies the degree of significance by which the former consistently deviates from the latter across each of the sliding windows. It tests the null hypothesis that the median of the probability distribution of the differences between the logarithm of the intensities from the test sample and those from the reference sample is zero. As a non-parametric test, Wilcoxon signed-rank test has low power when the sample size is small. To increase the test power, the user needs to use larger window sizes.

At the scoring step, Tilescope generates two tile maps, the signal map and the *P *value map (Figure 2c). Two values are calculated for each tile position: the pseudo-median of log-ratios, as a measure of the fold enrichment of the hybridization signal in the test sample over the reference at this genomic location and the probability, the *P *value, that the null hypothesis - the local intensities of the test and the reference samples are the same - is true. In a recent study of transcript mapping with high-density tiling arrays, Huber *et al*. [17] used a different approach to score tiles. Their method does not assess intensity difference at individual tiles. Instead, it tries to find a step function that best fits the log-ratio intensities along genomic coordinates.

### Feature identification

Given the tile map annotated with pseudo-medians and *P *values, Tilescope filters away tiles that are below user-specified thresholds. Retained tiles are used to identify either deferentially expressed genes or TFBSs. Currently, Tilescope users can choose one of three methods to identify such features (Figure 2d). The first method, 'max-gap and min-run', is a well-used method, initially used by Cawley *et al*. [25] to analyze their ChIP-chip tiling array data. The second method, 'iterative peak identification', is a new method that we developed to find genomic features iteratively. The third method, whose theoretical development is described in full elsewhere [27], effectuates file segmentation by using a 'hidden Markov model' (HMM) explicitly built on validated prior knowledge.

#### Max-gap and min-run

Based on the observation that a tile is usually too short to constitute a feature alone, the first method, modified from the scoring scheme used in Cawley *et al*. [25] and Emanuelsson *et al*. [28], groups together qualified tiles that are close to each other along the genomic sequence into 'proto-features' and then discards any proto-features that are too short. To use this method, a user needs to specify the maximum genomic distance ('max-gap') below which two adjacent qualified tiles can be joined and the minimum length ('min-run') of a proto-feature for it to be qualified as a feature.

#### Iterative peak identification

The second method, which we have recently developed and implemented as part of the pipeline, does not group tiles above thresholds into features. Instead, it identifies local signal 'peaks' in an iterative fashion. This method was developed to generate lists of non-overlapping features of a uniform genomic size.

Taking the signal map that has been generated in the tile scoring step using window-smoothing to integrate the data from multiple replicate arrays, this method first identifies the tile ('point source') that corresponds to the peak in the signal map with the global maximum signal that also meets a predefined *P *value threshold. A feature is then created centered at the genomic position of the peak with a predefined genomic size. We choose a feature size that is comparable with the average size of the fragmented ChIP DNA (typically about 1 Kb). The feature is assigned a signal measurement from the associated peak.

All tiles within a predefined distance from the located 'peak' are then removed from the signal map data. Typically, the distance is the same size as the selected features, though it can be larger. This is to ensure that apparent 'secondary peaks' in the signal maps that are really part of the same feature are not separately identified. The procedure is then iterated to find the next maximum 'peak' in the remaining signal map data. The iteration generates a list of features ranked by 'peak' signals and terminates when the identified 'peak' signal is below a specified signal enrichment threshold.

#### Hidden Markov model

The third method uses a supervised scoring framework based on HMMs to predict and score features in the genome tiled on the microarray [27]. Our method, based on a similar motivation as in [29], differs from previous HMM-based studies [30,31] by specifically considering validated biological knowledge (for example, experimental validation, gene annotation, and so on) and systematically incorporating it to score different types of array assays within the same framework.

For identification of transcriptionally active regions (TARs)/transcribed fragments (transfrags) in transcriptional tiling array data, a four-state (TAR, non-TAR, and two other intermediate transition states) HMM is constructed using the knowledge of gene annotation (information of the probes that fall into annotated gene regions). For ChIP-chip data, a two-state (TFBS and non-TFBS) HMM is constructed by using the knowledge of inner regions in genes to estimate the signal emission distribution *g*(*t*) of the non-TFBS state, and by using the subtraction of *g*(*t*) from the overall emission distribution *h*(*t*) to estimate the emission distribution *f*(*t*) of the TFBS state. In a more general ideal scenario, our framework first selects a medium-sized set of sub-regions by using some appropriate analysis methods (for example, the *MaxEntropy *sampling scheme discussed in [27]), and then utilizes the knowledge in these sub-regions as the training set to build the model for accurate analysis.

Further scoring on the initial analysis results can also be done by computing the posterior probabilities of each probe being active. The scores indicate the confidence in every single probe-level prediction and can be used to refine the previous analysis results by HMM. For instance, the identified active probes can be ranked according to the overall confidence levels in their regions and a threshold confidence level may either be set manually or be learned automatically to refine the original results.

#### Method comparison

**Figure 3:**
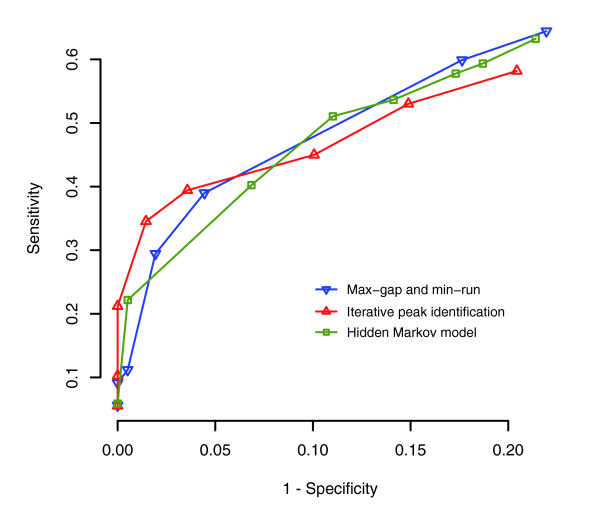
The ROC curves of the three feature identification methods implemented in Tilescope. The comparison of the performance of these methods was based on a well-studied STAT1 ChIP-chip data set and a list of experimentally tested STAT1 binding sites.

We compared the performance of these three feature identification methods using a well-studied STAT1 ChIP-chip data set. Composed of three technical replicates, this data set was used to identify a list of STAT1 binding sites in the ENCODE regions [27]. These sites were later experimentally tested. We analyzed this data set using Tilescope and generated three STAT1 binding site lists, each by a different feature identification method. Since identical tile scoring and thresholding parameters were used, the difference among these three lists reflects the underlying difference among the three feature identification methods. By using the list of the experimentally tested STAT1 binding sites, we were able to assess the sensitivity and specificity of each method. The receiver operating characteristic (ROC) curves in Figure 3 show that while, in general, the three feature identification methods implemented in Tilescope have similar performance (which can be measured by the area under the curve or other measurements such as the Matthews' correlation coefficient [32,33] and the minimum error rate [34]), the 'iterative peak identification' method is appreciably more sensitive at high (>95%) specificity.

## Conclusion

### Summary

Tilescope is an online software pipeline for processing high-density tiling microarray data. In a completely automated fashion, it will normalize signals between channels and across arrays, combine replicate experiments, score each array element, and identify genomic features. The program can process data from most gene expression, ChIP-chip, and arry-CGH (comparative genomic hybridization) experiments. Tilescope is designed with a graphical user-friendly interface to facilitate a user's data analysis task, and the results, presented in a clear, well organized manner on a web page, can be downloaded for further analysis.

### Future improvements

Tilescope is under active development: it is continually updated as better data processing methods become available.

## Availability

Tilescope is freely accessible for use at [35]. The source code of the pipeline is available at [36].

## Additional data files

The following additional data are available with the online version of this paper. Additional data file 1 includes the following supplementary material: a 'Data format optimization and standardization' section; a 'Technical details of the optimization algorithm' section; supplementary Table 1, 'The meaning of columns of various tab-delimited input data file formats'; supplementary Table 2, 'Attributes of the element tag defined in the configuration file'; and supplementary Table 3, 'File size reduction by Zip and our optimization algorithm'.

## Supplementary Material

Additional data file 1 Supplementary material: a 'Data format optimization and standardization' section; a 'Technical details of the optimization algorithm' section; supplementary Table 1, 'The meaning of columns of various tab-delimited input data file formats'; supplementary Table 2, 'Attributes of the element tag defined in the configuration file'; and supplementary Table 3, 'File size reduction by Zip and our optimization algorithm'. Click here for file
