# Supplementary material for: Tilescope: online analysis pipeline for high-density tiling microarray data
Source: Genome Biol. 2007 May 14;8(5):R81. doi: 10.1186/gb-2007-8-5-r81 (PMC1929149; doi:10.1186/gb-2007-8-5-r81)
Supplement: Additional data file 1 — Supplementary material: a 'Data format optimization and standardization' section; a 'Technical details of the optimization algorithm' section; supplementary Table 1, 'The meaning of columns of various tab-delimited input data file formats'; supplementary Table 2, 'Attributes of the element tag defined in the configuration file'; and supplementary Table 3, 'File size reduction by Zip and our optimization algorithm'. [file gb-2007-8-5-r81-S1.doc]

**Supplementary material**

*Data format optimization and standardization*

The tab-delimited text files generated by different image analysis software are exceptionally large in size and can have different ASCII formats. This raises several issues. First, large files require large disk storage space, long transmission time, and long file access time. Second, the result of a single microarray experiment could be specified by several files, such as both the design and the data files, which can be problematic to handle and organize. Third, various file formats require different programs to parse the data. Although currently several open specifications, like MIAME and MAGE-ML, are available to standardize the array data format, these standards usually neglect the data size issue and require users to provide information more than necessary and thus are cumbersome for regular data analyses.

Not only did we implement Tilescope to support various formats of the input data file—the GFF format, the PAIR format, the NimbleGen format (POS + GFF/PAIR), and the Affymetrix format (BPMAP + CEL), but also developed a new algorithm (Supplementary Table 1-3) that can reduce the physical data file size, handle the data set in an organized manner, and enhance the performance of Tilescope. In order to reduce the data size, we store the data values as their primitive types rather than in ASCII format. For example, an integer ‘147,971,601’ will occupy 9 bytes in an 8-bit ASCII format but only 4 bytes in our format. Another example is the nucleotide sequence. A sequence such as ‘TCGAGGCCTTAAGCTCTTGAGAGGT’ will occupy 25 bytes in ASCII but only 7 bytes in our format since we use two bits to represent each base. After optimizing the data file representation, we compress all the files, including the meta-files storing the information describing those data files, into one single archive file.

Our bit optimization approach not only achieves significant file size reduction but also enables us to efficiently locate by random access a particular record for a certain set of coordinates when necessary. This is because we store every entry in a fixed number of bits and sort all the entries by their coordinates, so that the offset of the record in the byte stream can be easily calculated given that all the data are in contiguous coordinates. Moreover, an even higher compression rate can be obtained if a ‘deflate’ algorithm (see http://www.rfc-editor.org/rfc/rfc1951.txt) is applied after the array data is optimized than it is not (Supplementary Table 3). This is especially advantageous for file transfer over the network.

Since our algorithm allows us to select which data columns in the text files to be included, it omits any unnecessary information in the original data set and consequently further reduces the size of the file to be transmitted. This capability, together with the aforementioned improvements, can reduce the size of the original data set by up to 90%. This size reduction greatly reduces the amount of time required to transmit the data from the client computer to our pipeline server over the Internet. To ensure the consistency of the data file processing, we convert the archived data files into the standardized GFF format on the pipeline server. As a result, the data files can always be processed in a consistent manner.

*Technical details of the optimization algorithm*

Nearly all the data files and design files of microarray are tab delimited. Generally speaking, each line in the files represents a data record and each tab delimited field represents a particular type of data (see Supplementary Table 1). For example, the first column of the data in a GFF file is a sequence name. Since different columns represent different data types, the data value in a column could be a string, number, decimal, nucleotide sequence, or etc. For example, the sixth column, which represents the X coordinate of a microarray, in a PAIR file is in number format. Due to the fact that these data types are already known in each format, we could take this assumption and optimize the data storage according to their types. As mentioned before, storing the data in ASCII format makes the file larger than it should be if compared to storing the data in their primitive types or any other optimal types. For instance, a 32 bit integer requires 10 bytes in ASCII but only 4 bytes in its primitive type. A more extreme case would be the nucleotide sequence. Since a nucleotide of DNA can only be A, T, C, or G, we actually only need to use 2 bits to store each nucleotide so that one byte can already store 4 nucleotides.

As a result, our optimization algorithm mainly focuses on converting these kinds of data into a more optimal format. To achieve this, we created a configuration file containing the information of the data column for different microarray file formats (see Supplementary Table 2). We also created a set of Java API which parses these files and optimizes the data according to the configuration file. We found that the size of the optimized data is reduced significantly if compared to the original size although the compression ratio is not as good as Zip. However, we also found that a higher compression ratio can be achieved if we zip the optimized data instead of the raw data (see Supplementary Table 3). To this end, we chose to first optimize our input data using our optimization algorithm and then bundle the optimized data with its configuration file using zip. This helps reducing the overall file size substantially and so the transfer time. Therefore, our optimization algorithm is not replacing any compression algorithm but enhancing the compression ratio. An added advantage of our algorithm is that it enables us to locate a data record more easily. This is because we store every data entry in a fixed number of bits and sort all the entries by their coordinates, so that the offset of a particular record in the byte stream can be easily calculated by their coordinates given that all the data are in contiguous coordinates. Although our format is capable of locating a record faster, we instead chose to convert this “middle” format into the standard GFF format for processing independent of the original format of the data. This ensures our processing consistency as we only need to deal with a single format and the most important reason is that we will be able to give the users back their data in a standard format later if necessary.

| **Supplementary Table 1** The meaning of columns of various tab-delimited input data file formats. | | | | | | | | | |
| --- | --- | --- | --- | --- | --- | --- | --- | --- | --- |
| 1 | 2 | 3 | 4 | 5 | 6 | 7 | 8 | 9 | 10 |
| *GFF format* | | | | | | | | | |
| Seq. Name | Source | Feature | Start Pos. | End Pos. | Score | Strand | Frame | Group | — |
| *PAIR format* | | | | | | | | | |
| Image ID | Gene Exp. Opt. | Seq. ID | Probe ID | Pos. | X | Y | Match Index | Perfect Match | Mis-match |
| *POS format* | | | | | | | | | |
| Seq. ID | Seq. Name | Position | Probe ID | Count | Length | — | — | — | — |
| *BPMAP format* | | | | | | | | | |
| X | Y | Seq. Name | Pos. | Probe Seq. | — | — | — | — | — |
| *CEL format* | | | | | | | | | |
| X | Y | Mean | SD | No. of Pixels | — | — | — | — | — |
| Note: We assume all the data files, including the design files if any, are in tab delimited format. The data type of each column of data of each format is defined in an XML configuration file. In the configuration file, it also stores the file type, namely ‘design’ and ‘data’, of each supported format. | | | | | | | | | |

| **Supplementary Table 2** Attributes of the element tag defined in the configuration file ^§^. | | | | |
| --- | --- | --- | --- | --- |
| Type | Minimum value | Maximum value | Decimal | Size (example) |
| Number | The minimum possible integer | The maximum possible integer | — | 4 bytes (147,971,601) |
| Decimal | The minimum possible integer | The maximum possible integer | Number of decimal places | 3 bytes (65535.99) |
| Text | Unused (0) | The maximum number of characters | — | 6 bytes (N10023) |
| Nucleotide Sequence | Unused (0) | The number of bases in the sequence | — | 7 bytes ^†^ |
| Highly  Repetitive  String | Unused (0) | The maximum possible number of unique strings | — | 1 byte ^‡^ |
| § ‘Type’, ‘Min’, ‘Max’, and ‘Decimal’ are the attributes that define the data type of a column in a data file.  †. A string of 25 bases (e.g., AAACGAATTGCCATTAGGCCATTAG)  ‡. 256 unique strings.  Note: Following is an example of the definition of the Affymetrix design file format in the configuration file:  <dataset type="design" format="txt">  <elements>  <element name="x" min="0" max="65535" type="number" disabled="false" />  <element name="y" min="0" max="65535" type="number" disabled="false" />  <element name="seq" min="0" max="255" type="hrString" disabled="false" />  <element name="pos" min="0" max="2147483647" type="number" disabled="false" />  <element name="probe" min="0" max="25" type="sequence" disabled="false" />  </elements>  </dataset>  The ‘name’ attribute is the column identifier used in the API to retrieve back the data corresponding to that column. The 'disabled' attribute indicates if the data of that column should be converted. | | | | |

| **Supplementary Table 3** File size reduction by Zip and our optimization algorithm. ^§, †^ | | | | | | | |
| --- | --- | --- | --- | --- | --- | --- | --- |
|  | | Design File | | Data File | | All Files | |
| *Affymetrix*: | |  | |  | |  | |
|  | Original | 36 |  | 37.5 |  | 73.5 |  |
|  | Zip | 11.9 |  | 7.7 |  | 19.6 |  |
|  | Optimization | 11.5 |  | 10.9 |  | 22.5 |  |
|  | Optimization+Zip | — |  | — |  | 12.1 | 83.5% ^‡^ |
| *NimbleGen*: | |  | |  | |  | |
|  | Original | 16.4 |  | 32.4 |  | 48.8 |  |
|  | Zip | 2.3 |  | 6.1 |  | 8.4 |  |
|  | Optimization | 10.6 |  | 5.8 |  | 16.4 |  |
|  | Optimization+Zip | — |  | — |  | 6.8 | 86.1% ^‡^ |
| §. This table compares the size of two different sets of sample input files, an Affymetrix data set and a NimbleGen data set, before and after optimization and zip. “Original”, “Zip”, and “Optimization” represent the original, zipped, and optimized size of the data respectively. “Optimization+Zip” represents the size of the data zipped after optimization.  †. File size is in MB.  ‡. Percentage of total file size reduction. | | | | | | | |
